# Immunosuppressive Drugs and COVID-19: A Review

**DOI:** 10.3389/fphar.2020.01333

**Published:** 2020-08-28

**Authors:** Tessa S. Schoot, Angèle P. M. Kerckhoffs, Luuk B. Hilbrands, Rob J. van Marum

**Affiliations:** ^1^ Department of Nephrology, Radboud University Medical Center, Nijmegen, Netherlands; ^2^ Department of Nephrology, Jeroen Bosch Hospital, ‘s-Hertogenbosch, Netherlands; ^3^ Department of Geriatric Medicine Jeroen Bosch Hospital, ‘s-Hertogenbosch, Netherlands; ^4^ Department of Clinical Pharmacology, Jeroen Bosch Hospital, ‘s-Hertogenbosch, Netherlands; ^5^ Department of Medicine for Older People, VU University Medical Center, Amsterdam, Netherlands

**Keywords:** COVID-19, SARS-CoV-2, coronavirus, immunosuppressive drugs, corticosteroids, mycophenolic acid, calcineurin inhibitors, mTOR inhibitors

## Abstract

**Background:**

Coronavirus disease 2019 (COVID-19) is caused by severe acute respiratory syndrome coronavirus 2 (SARS-CoV-2). It is currently unknown whether immunosuppressive drugs are advantageous or detrimental in patients with COVID-19. Immunosuppressive drugs could be harmful in the initial phase of COVID-19. In this phase, the host immune response is necessary to inhibit viral replication. However, immunosuppressive drugs might have a beneficial effect in the later, more severe phase of COVID-19. In this phase, an overshoot of the host immune response (the “cytokine storm”) can cause ARDS, multiorgan failure and mortality.

**Aim:**

To summarize the available evidence on the effect of immunosuppressive drugs on infection with SARS-CoV-2. The effects of immunosuppressive drugs on similar pandemic coronaviruses may resemble the effects on SARS-CoV-2. Thus, we also included studies on the severe acute respiratory syndrome coronavirus (SARS-CoV) and Middle East respiratory syndrome coronavirus (MERS-CoV)

**Methods:**

The study protocol was registered in PROSPERO (registration number CRD42020181137). We included randomized controlled trials (RCTs), cohort studies with a control group and case-control studies concerning humans ≥ 18 years old. We also included *in-vitro* studies and animal studies with a control group.

**Results and Conclusion:**

Sixty-nine studies were included. Interestingly, MPA inhibits SARS-CoV-2 replication *in-vitro*. Clinical studies are needed to confirm the inhibitory effect of MPA on SARS-CoV-2 replication *in-vivo*. There are indications that corticosteroids and IL-6 inhibitors, like tocilizumab, can reduce mortality and prevent mechanical ventilation in patients with COVID-19. However, observational studies have contradictory results and the risk of bias is high. Thus, these results have to be confirmed in high-quality clinical trials before these drugs can be implemented as standard care. Based on the positive results of CNIs, mTOR inhibitors and thiopurine analogues in *in-vitro* studies with SARS-CoV and MERS-CoV, it would be interesting to investigate their effects on SARS-CoV-2 replication.

## Introduction 

Coronavirus disease 2019 (COVID-19), caused by severe acute respiratory syndrome coronavirus 2 (SARS-CoV-2), has a mild course in the majority of patients. However, approximately 14% of the patients require hospitalization for oxygen support and 5% is admitted to the intensive care unit (ICU) (World Health Organization (WHO), 2020).

COVID-19 has a triphasic course (Siddiqi and Mehra, 2020). In the first phase, patients have mild respiratory and systemic symptoms such as dry cough, malaise and fever. An adequate response of the innate and adaptive immune system can eliminate the virus and preclude disease progression to the next, more severe stages (Shi et al., 2020). In the second phase, viral multiplication and localized inflammation of the lung tissue occurs, causing viral pneumonia. A minority of patients with COVID-19 will undergo a transition into the third and most severe phase of illness: a syndrome of systemic hyperinflammation, also referred to as secondary hemophagocytic lymphohistiocytosis or the cytokine storm syndrome. These patients have high levels of pro-inflammatory cytokines, such as interleukin (IL)-2, IL-6, IL-7 and tumor-necrosis factor-α (TNF-α) (Huang et al., 2020; Zhang et al., 2020). In this phase, patients can develop acute respiratory distress syndrome (ARDS) and multiorgan failure, which are the main causes of mortality of COVID-19 (Cao et al., 2020; Du et al., 2020; Mehta et al., 2020; Ruan et al., 2020; Wu et al., 2020).

It is hypothesized that immunosuppressive drugs could be used to prevent and treat the hyperinflammatory phase of COVID-19. However, these drugs inhibit the host immune response against the virus as well. Thus, they could be harmful in earlier stages of COVID-19. The net effect of immunosuppressive drugs in patients with COVID-19 is currently subject of debate.

The primary aim of this review is to summarize the available evidence on the effect of immunosuppressive drugs on infection with SARS-CoV-2. Given the short time period since the start of the COVID 19 pandemic, relatively few studies on COVID-19 are completed. Therefore, we also searched for papers reporting the effect of immunosuppressive drugs on infection with severe acute respiratory syndrome coronavirus (SARS-CoV) and Middle East respiratory syndrome coronavirus (MERS-CoV), two other pandemic coronaviruses belonging to the Nidovirales order (de Wit et al., 2016; Huang et al., 2020). These viruses are the causative agents of severe acute respiratory syndrome (SARS) and Middle East respiratory syndrome (MERS), which share many features with COVID-19 (Wang et al., 2004; Yin and Wunderink, 2018; Wang D. et al., 2020). Thus, the effects of immunosuppressive drugs on these viruses may resemble the effects on SARS-CoV-2. The results of this review can be used in the development of evidence-based guidelines for the management of patients with COVID-19 and of patients using immunosuppressive drugs during the COVID-19 pandemic.

## Methods

We conducted a PubMed search, without limitations for the publication date or publication type. In the search, the terms “COVID-19”, “SARS”, “MERS” (and multiple synonyms) were combined with search terms for the different classes of immunosuppressive drugs (see: **Supplementary File 1**). The last search was performed on 8 July 2020. All papers were screened by title and abstract, and the full text of potentially eligible papers was read. In addition, the reference list of all identified papers was screened manually. To be included in our review, studies had to meet the following inclusion criteria:

1. Studies concerning humans ≥ 18 years old, animals or cells infected with SARS-CoV-2, SARS-CoV or MERS-CoV.
2. *In-vitro* or *in-vivo* treatment with any of the following immunosuppressive drugs: calcineurin inhibitors (CNIs; cyclosporine (CsA), tacrolimus(TAC)), antimetabolites (like mycophenolic acid (MPA), azathioprine (AZA), methotrexate), mammalian target of rapamycin (mTOR) inhibitors (sirolimus, everolimus), corticosteroids (like methylprednisolone, hydrocortisone, prednisone, dexamethasone), cyclophosphamide, rituximab, alemtuzumab, IL-6 inhibitors (like tocilizumab), basiliximab, anakinra, dupilumab, brodalumab, secukinumab, ixekizumab, anti-TNF-α inhibitors (like infliximab), abatacept, belatacept, or eculizumab.
3. Data on one or more of the following outcome measures: viral load, viral replication, clinical outcome (e.g. mortality rate, ICU admission rate, length of hospital stay).
4. Study type: *in-vitro* study, animal study with control group, randomized controlled trial (RCT), cohort study with control group, case-control study.
5. Language: English.

We categorized the obtained information per immunosuppressive drug class. In addition, we sorted the data according to the type of outcome parameter, i.e. viral load or clinical outcome. The study protocol was registered in PROSPERO (registration number CRD42020181137).

## Results

The database search yielded 1939 search hits, with 69 studies matching the inclusion criteria. A summary of the results is presented in **Table 1**.

**Table 1:** Summary of results.

Immunosuppressive drug	Virus	Number of studies			Summary of results	
		*In-vitro*	Animal	Human	Viral load and viral replication	Clinical outcome
**Corticosteroids**	SARS-CoV-2	0	0	18	Cohort studies have different results. 5 cohort studies found that steroid use had no effect on SARS-CoV-2 clearance time. 3 cohort studies report that steroid use associated with longer SARS-CoV-2 clearance time. All studies have a high risk of confounding (by indication).	One RCT found that dexamethasone use was associated with lower 28-day mortality rate, shorter length of hospital stay and lower prevalence of mechanical ventilation. Limitations: open-label. Observational studies have different results: 6 cohort studies found that steroids have a beneficial effect, 2 cohort studies conclude that steroids have a detrimental effect and 1 cohort studies report that steroids have no effect on mortality. The risk of confounding (by indication) is high in all studies. 3 cohort studies report that higher steroid dose is associated with death, but they have a significant risk of confounding by indication.
	SARS-CoV	0	0	18	One RCT found that steroid use had no effect on SARS-CoV clearance time, but the risk of confounding was high. One autopsy study found that steroid use was not associated with viral load in lung tissue, but this study may have been underpowered.	Cohort studies have different results: 2 cohort studies found that steroids have a beneficial effect, 7 cohort studies conclude that steroids have a detrimental effect and 3 cohort studies report that steroids have no effect on mortality. Moreover, the risk of confounding (by indication) is high in all studies. Neither can we draw conclusions on the ideal timing of steroid administration or the ideal steroid dose. Cohort studies report conflicting results and have high risk of confounding (by indication).
	MERS-CoV	0	0	2	One cohort study in ICU patients found that steroid use was associated with longer MERS-CoV clearance time. There is a high risk of confounding (by indication).	One cohort study found that steroid use was associated with higher mortality. The risk of bias could not be assessed because of incomplete baseline characteristics. In 1 cohort study in ICU patients had no clear conclusion, because several statistical methods provided different results.
**Calcineurin inhibitors**	SARS-CoV-2	0	0	0	*No evidence available*	
	SARS-CoV and MERS-CoV	6	0	0	CsA inhibits the replication of SARS-CoV and MERS-CoV *in-vitro*. TAC inhibits the replication of SARS-CoV *in-vitro*.	*No evidence available*
**Mycophenolic acid**	SARS-CoV-2	2	0	0	MPA inhibits SARS-CoV-2 replication *in-vitro*.	*No evidence available*
	SARS-CoV	2	0	0	MPA does not inhibit the proteolytic activity of SARS-CoV PL^pro^ or SARS-CoV replication *in-vitro*.	*No evidence available*
	MERS-CoV	5	1	1	MPA inhibits the proteolytic activity of MERS-CoV PL^pro^ or MERS-CoV replication *in-vitro*.	One cohort study found that MMF use was associated with a lower mortality rate, but there is significant risk of confounding by indication.
**Thiopurine analogues**	SARS-CoV-2	0	0	0	*No evidence available*
	SARS-CoV	3	0	0	6MP and 6TG inhibit the proteolytic activity of MERS-CoV and SARS-CoV PL^pro^.	*No evidence available*
	MERS-CoV	0	0	0	*No evidence available*
**mTOR inhibitors**	SARS-CoV-2	0	0	0	*No evidence available*	
	SARS-CoV	0	0	0	*No evidence available*	
	MERS-CoV	1	0	0	Sirolimus and everolimus reduce MERS-CoV titers *in-vitro*.	*No evidence available*
**Anti-TNF-α agents**	SARS-CoV-2	0	0	0	*No evidence available*	
	SARS-CoV	0	1	0	*No evidence available*	In an animal study, administration of anti-TNF-α monoclonal antibody had no effect on the mortality rate, but the onset of symptoms was somewhat delayed.
MERS-CoV		0	0	0	*No evidence available*	
**Anakinra**	SARS-CoV-2	0	0	1	*No evidence available*	In one cohort study in patients with COVID-19, ARDS and hyperinflammation, anakinra use was associated with a lower 3-week mortality rate, but a longer duration of mechanical ventilation. The study has a significant risk of confounding.
	SARS-CoV	0	0	0	*No evidence available*	
	MERS-CoV	0	0	0	*No evidence available*	
**Tocilizumab and other IL-6 inhibitors**	SARS-CoV-2	0	0	9	*No evidence available*	One retrospective cohort study found that treatment with tocilizumab in patients with COVID-19 is associated with a higher mortality rate, but this study has a high risk of confounding. Four observational studies found no effect of tocilizumab or sarilumab in patients with COVID-19. Four other retrospective cohort studies found that mortality and ICU admission rate were lower in patients treated with tocilizumab compared to controls.
	SARS-CoV	0	0	0	*No evidence available*	
	MERS-CoV	0	0	0	*No evidence available*	

6MP, 6-mercaptopurine; 6TG, 6-thioguanine; ARDS, acute respiratory distress syndrome; AZA, azathioprine; CNI, calcineurin inhibitor; COVID-19, coronavirus disease 2019; CsA, cyclosporin A; MERS, Middle East respiratory syndrome; MMF, mycophenolate mofetil; MPA, mycophenolic acid; mTOR, mammalian target of rapamycin; SARS-CoV, severe acute respiratory syndrome coronavirus; TAC, tacrolimus; TNF-α, tumor-necrosis-factor-α.

### Corticosteroids

Corticosteroids have a wide range of anti-inflammatory and immunomodulatory effects, including inhibition of the synthesis of pro-inflammatory cytokines, reduction of leucocyte trafficking, and induction of apoptosis of T-lymphocytes (Lansbury et al., 2019). In addition, corticosteroids might increase the sensitivity to vasopressors (Lansbury et al., 2019) and can be used to treat adrenal insufficiency, which is present in 20% of the patients with critical illness, and in up to 60% of those with sepsis and septic shock (Hui et al., 2018).

In **Table 2**, the 38 clinical studies matching the inclusion criteria are summarized.

**Table 2:** Overview of clinical studies of corticosteroids in the treatment of patients with COVID-19, SARS and MERS.

Paper (author, year)	Country	Study design	Subjects (n)	Subjects using steroids (n)	Inclusion criteria	Type of steroid	Dosage	Main conclusion on antiviral effect	Main conclusion on clinical outcome	Risk of bias and commentary
**COVID-19**										
Cao et al. (2020)	China	Cohort study	102	51	Hospitalized patients with COVID-19	Methyl-pred	UNK	NA	The rate of glucocorticoid treatment was not significantly different between surviving and deceased patients (64.7% and 47.1% respectively, p=0.184).	Risk of confounding (by indication). Not entirely clear if data was collected prospectively or retrospectively.
Chen et al. (2020)	China	Retrospective cohort study	267	29	Hospitalized patients with COVID-19	UNK	UNK	Median duration of viral shedding was longer in patients treated with steroids (18.0 vs. 12.0 days, p<0.001). In multivariate analysis, steroid use was associated with prolonged viral RNA shedding (HR 0.60, 95% CI 0.39-0.94).	NA	Risk of confounding (by indication)
Fadel et al. (2020)	USA	Retrospective cohort study	219	132	Hospitalized patients with COVID-19, bilateral pulmonary infiltrates and oxygen requirement	Methyl-pred	0.5 – 1 mg/kg/day for 3 – 7 days	NA	Mortality, mechanical ventilation rate, prevalence of ARDS, ICU admission rate and length of hospital stay were all statistically significantly lower in patients in the steroid-group than in controls.	Risk of confounding (by indication), e.g. because the same proportion of patients in both groups eventually used steroids (56.8% vs. 68.2%, p = 0.094). Selection bias: patients who died or were discharged within 24 h of admission were excluded.
Fang et al. (2020)	China	Retrospective cohort study	78	25	Hospitalized patients with COVID-19 with mild vs severe illness	Methyl-pred	Average daily dose 38 vs 40 mg. Total 160 vs 280 mg.	The time from admission to first negative PCR was not significantly different between patients with and without steroid treatment.	NA	Risk of confounding (by indication).
Fernández Cruz et al. (2020)	Spain	Retrospective cohort study	463	396	Hospitalized patients with COVID-19 pneumonia, and ARDS or hyperinflammation	Methyl-pred	1mg/kg/day with or without pulses (<250, 250 or 500 mg/day)	NA	In-hospital mortality was lower in patients treated with steroids than in controls (HR 0.51, 95% CI 0.27 – 0.96). The NNT was 10. Steroid use was an independent predictive factor for in-hospital mortality in multivariate analysis.	Risk of confounding, although this was partly corrected by using propensity score matching in the multivariate analysis,
Giacobbe et al. (2020)	Italy	Retrospective cohort study	78	24	Patients with COVID-19 admitted to the ICU for >48 h	Methyl-pred	UNK	NA	Use of methylpred was independently associated with development of a blood stream infection in multivariable analysis (HR 3.95, 95% CI 1.20 – 13.03).	Risk of confounding. Complete assessment of risk of bias is not possible as essential information is missing, such as baseline characteristics of subgroups.
Horby et al. (2020)	UK	RCT (RECOVERY trial)	4321	2104	Hospitalized patients with COVID-19	Dexa	6 mg daily for max. 10 days (median 6 days)	NA	The 28-day mortality rate was lower in dexa-treated patients than controls (21.6% vs. 24.6%; age-adjusted RR 0.83, 95% CI 0.74-0.92), also in the subgroups of patients requiring oxygen (RR 0.80, 95% CI 0.70-0.92) or invasive mechanical ventilation (RR 0.65, 95% CI 0.51-0.82). In the subgroup of patients that were not treated with any type of respiratory support at randomization, the 28-day mortality rate was not different for dexa treated patients and controls (RR 1.22, 95% CI 0.93-1.61). The rate of invasive mechanical ventilation was lower in dexa-treated patients than controls (RR 0.76, 95% CI 0.61-0.96). The length of hospital stay was shorter for dexa-treated patients and controls (median 12 vs. 13 days), and a greater proportion was discharged after 28 days.	Risk of confounding (by indication): very low. Small risk of selection bias: dexa was not available in the hospital for some eligible patients.
Li et al. (2020)	China	Retrospective cohort study	548	341	Hospitalized patients with COVID-19	UNK	Median cumulative dose during admission 200 mg	NA	Steroid use was higher in patients who required mechanical ventilation than in patients who did not (34.1% vs 12.2%). High-dose steroids (≥ 1 mg/kg/day pred equivalent) use was a risk factor for death. Low-dose steroid use was not.	Risk of confounding (by indication), although this is partly corrected by using a prospensity score in the multivariate analysis.
Li et al. (2020)	China	UNK	206	UNK	Patients with COVID-19	UNK	High-dose (80 mg/day) or low-dose (40 mg/day)	Cox regression model analysis showed that high-dose steroids was associated with prolonged viral shedding (aHR = 0.67, 95% CI 0.46 - 0.96), but low-dose steroids was not (aHR 0.72, 95% CI 0.48 1.08).	NA	The risk of bias cannot be assessed. Study design, inclusion and exclusion criteria are not stated. Method of statistical analysis not clearly described, e.g. not stated how aHR was calculated.
Ling et al. (2020)	China	Retrospective cohort study	66	5	Patients recovered from COVID-19 (resolution of symptoms and negative PCR)	UNK	UNK	Duration of viral RNA detection was significantly longer in steroid-treated patients than in steroid-free patients: for throat swabs 15 days vs 8 days, and for feces 20 days vs 11 days.	NA	Risk of confounding (by indication).
Lu et al. (2020)	China	Retrospective cohort study	244	151	Hospitalized patients with COVID-19, with ARDS or sepsis with acute organ dysfunction	UNK	Median hydrocort-equivalent dose 200 mg/day (range 100–800)		In multivariate analysis including propensity score matching, steroid use was not associated with higher overall 28-day mortality (aOR 1.05, 95% CI 0.15–7.46). Higher steroid dose was associated with higher mortality (aHR per 10 mg dose increase 1.04, 95% CI 1.01–1.07).	Risk of confounding (by indication), although this is partly corrected by using prospensity score matching in the multivariate analysis.
Mo et al. (2020)	China	Retrospective cohort study	155	79	Hospitalized patients with COVID-19 pneumonia	UNK	UNK	NA	The 70 patients (45%) who reached clinical and radiological remission within 10 days were less likely to have received corticosteroids than patients who did not reach remission.	Risk of confounding (by indication).
Sanz Herrero et al. (2020)		Retrospective cohort study	72	56	Hospitalized patients with COVID-19 treated with tocilizumab	Methyl-pred	250 mg once, then 40 mg twice daily for 4 days		Methylpred use was associated with a lower risk of death (HR 0.20, 95% CI 0.08 – 0.47).	Risk of confounding (by indication). Limited generalizability (subgroup of patients treated with tocilizumab).
Wang et al. (2020)	China	Retrospective cohort study	46	26	Hospitalized patients with COVID-19	Methyl-pred	1-2 mg/kg/day		In patients treated with methylpred, mechanical ventilation was less prevalent, length of ICU and total hospital stay were shorter, and CRP and IL-6 decreased faster than in patients not treated with steroids. The mortality rate was not significantly different.	Risk of confounding (by indication).
Wu et al. (2020)	China	Retrospective cohort study	201	11	Hospitalized patients with COVID-19	Methyl-pred	UNK	NA	In the 41.8% of the patients with ARDS, methylpred use was associated with lower risk of death (HR 0.38).	Risk of confounding (by indication).
Xu et al. (2020)	China	Retrospective cohort study	113	64	Hospitalized patients with COVID-19	Methyl-pred	0.5-1 mg/kg/day	Steroid use was more prevalent in patients with still detectable viral RNA ≥ 15 days after start of symptoms than in patients with a negative PCR within 15 days (64.5% vs. 40.5%), but steroid use was not an independent risk factor for prolonged viral RNA detection.	NA	Risk of confounding (by indication). Different specimens (e.g. bronchoalveolar lavage fluid vs. nasopharyngeal swab) were used for PCR, which influences sensitivity.
Yuan et al. (2020)	China	Retrospective cohort study	132	35	Patients with non-severe COVID-19 pneumonia	Methyl-pred	Max. dose: median 52.5 mg (IQR 40-50 mg)	The time until negative SARS-CoV-2 PCR was not significantly different between steroid-treated patients and controls (median 20.3 vs. 19.4 days, p = 0.067).	The percentage of patients with disease progression was not statistically different between both groups (11.4% steroid-treated patients vs. 2.9% controls), and neither was duration of hospital stay and duration of fever.	Risk of confounding. Selection bias: only patients that could be matched to a control by propensity score were included.
Zha et al. (2020)	China	Retrospective cohort study	31	11	Hospitalized patients with COVID-19	Methyl-pred	40-80mg/day for median 5 days	The viral clearance time did not differ between steroid-treated and steroid-free patients.	Duration of symptoms and length of hospital stay were the similar for steroid-treated and steroid-free patients.	Risk of confounding (by indication).
Zheng et al. (2020)	China	Retrospective cohort study	55	21	Hospitalized patients with COVID-19	Methyl-pred	0.5-1 mg/kg/day	The rate of SARS-CoV-2 clearance and SARS-CoV-2 antibody formation did not differ between steroid-treated and steroid-free patients.	Recovery of radiologic images was not different in steroid-treated patients as compared to steroid-free patients.	Risk of confounding (by indication).
**MERS**										
(Arabi et al. 2018)	Saudi Arabia	Retrospective cohort study	309	151	Patients with MERS admitted to the ICU	Hydro-cort Methyl-pred Dexa Pred	UNK	In univariate analysis, MERS-CoV RNA clearance did not differ between steroid-treated and steroid-free patients. In a marginal structural model, corticosteroid use was associated with a delay in MERS coronavirus RNA clearance (adjusted HR 0.35; 95% CI 0.17–0.72).	In a multivariable logistic regression model, steroid use was associated with higher 90-day mortality (adjusted OR 1.87, 95% CI 1.02-3.44), but not in a Cox proportional hazard model and a marginal structural model.	The statistical models may not fully account for all confounders.
(Alfaraj et al. 2019)	Saudi Arabia	Retrospective cohort study	314	UNK	Hospitalized symptomatic MERS patients	UNK	UNK	NA	Corticosteroid use was associated with higher mortality in binary logistic regression (OR 3.85, 95% CI 1.95 – 7.57).	Assessment of bias is not possible as essential information is missing, such as baseline characteristics.
**SARS**										
(Ho et al. 2003)	China	Retrospective cohort study	72	72	Patients with SARS treated with steroids	1. Methyl-pred 2. Hydro-cort	1. 2-3 mg/kg/day or ≥ 500 mg/day 2. Median 480 mg/day	NA	Patients treated with low-dose steroids had worse chest radiographic scores on day 14 and 21, and more frequently needed supplemental oxygen therapy than patients treated with high-dose steroids. The rate of ICU admission, mechanical ventilation and mortality after 3 weeks was not significantly different between both groups	Risk of confounding. The cumulative steroid dose on day 21 was not different between the low-dose and high-dose group as many patients were treated with additional high-dose methylpred.
Zhao et al. (2003)	China	Prospective cohort study	190	UNK	Hospitalized patients suspected SARS, without pulmonary comorbidity	Methyl-pred	80-160 mg/day or 160-1000 mg/day	NA	All 60 patients who were treated with methylpred when fever persisted ≥ 3 days recovered without need of mechanical ventilation. In patients treated with methylpred when symptoms or radiological abnormalities worsened, 33% was treated with mechanical ventilation and 12% died of ARDS. In patients who were only treated with methylpred if they had not recovered after 14 days, 7% needed mechanical ventilation and 6% died.	Assessment of risk of confounding is not possible (disease severity and baseline patient characteristics are not provided). Patients were not randomly allocated to a treatment protocol, and adherence to treatment protocol seems not strict. It is not clearly stated how many patients were treated with steroids. SARS was not confirmed with PCR or serology.
Gomersall et al. (2004)	China	Retrospective cohort study	54	54	Patients with SARS admitted to the ICU	UNK	UNK	NA	Lower mean daily steroid dosage was associated with the composite endpoint (death and/or mechanical ventilation at 28 days) in univariate analysis.	Risk of confounding (by indication). Difference in mean corticosteroid dose seems coincidental and not clinically relevant (173 vs. 163 mg/day).
Jang et al. (2004)	Taiwan	Cohort study	29	21	Hospitalized patients suspected of SARS	UNK	UNK	NA	Duration between start of steroids and onset of symptoms was not associated with ICU admission and need of ventilatory support in univariate analysis. Six out of seven patients with nosocomial infections had received pulse steroid therapy.	Risk of confounding. 13 patients had a negative SARS-CoV PCR. Not entirely clear if data was collected prospectively or retrospectively.
Mazzulli et al. (2004)	Canada	Retrospective cohort study	11	6	Deceased patients with SARS	UNK	UNK	Steroid use was not associated with viral load in lung tissue (in 5 patients <10^6^ copies/g lung tissue, 1 patient > 10^6^ copies/g lung tissue, p = 0.08) in univariate analysis.	NA	Risk of confounding. Small sample size.
Ng et al. (2004)	China	Prospective cohort study	57	48	Adult patients recovered from SARS	UNK	UNK	NA	Pulse steroid use was independently associated with the presence of CT abnormalities during follow-up in multivariate analysis (OR 6.65, 95% CI 1.06-41.73).	Assessment of confounding (by indication) is not possible (baseline characteristics and disease severity are not provided). Timing of follow-up CT is not mentioned. It is not stated whether patients were treated with low-dose steroids.
Sung et al. (2004)	China	Prospective cohort study	138	138	Hospitalized patients suspected of SARS	1. Pred 2. Hydro-cort 3. Methyl-pred	1. 0.5-1mg/kg/day 2. 300mg/day 3. 0.5g/day for 3-6 days	NA	25 patients (18%) had a sustained or partial response after treatment with oral pred or intravenous hydrocort; the remaining 113 patients had no response. Treatment of 107 patients with methylpred resulted in sustained or partial response in 95 patients (89%).	Risk of confounding. SARS-CoV infection was not confirmed in 6 patients.
Auyeung et al. (2005)	China	Retrospective cohort study	78	66	Hospitalized adults suspected of SARS	UNK	UNK	NA	ICU admission and mortality did not differ between steroid-treated and steroid-free patients. In multivariate analysis, steroid use was associated with higher risk of ICU admission and mortality (adjusted OR 20.7, 95% CI 1.3 – 338.0).	Risk of confounding (by indication). In only 61 patients SARS was laboratory confirmed.
Chen et al. (2005)	Taiwan	Cohort study	67	44	Hospitalized patients suspected of SARS	UNK	UNK	NA	The proportion of patients treated with steroids did not differ between those with and without ARDS.	Risk of confounding (by indication). It is not clear whether steroids were administered before development of ARDS, or as treatment of ARDS. Only 33% had a positive PCR and only 85% anti-SARS-CoV antibodies. Not entirely clear if data was collected prospectively or retrospectively.
Hui et al. (2005)	China	Prospective cohort study	110	UNK	Patients with SARS discharged from hospital	UNK	Mean total dose (hydrocort equivalent) 18.9g for ICU patients and 8.2g for non-ICU patients	NA	Total dose of steroid was not associated with the result of the 6-minute walking test at 3 and 6 months. A higher total steroid dose was associated with more severe radiographic abnormalities at 6 months.	Assessment of risk of confounding (by indication) is not possible (disease severity and many relevant baseline characteristics are not provided)
Lee et al. (2004)	China	RCT (hydrocort vs. placebo)	17	16	Hospitalized patients with SARS admitted within 6 days of symptom onset, without respiratory failure at admission.	1. Hydro-cort 2. Methyl-pred	Total daily dose 1. 300mg 2. 500mg	The time until SARS-CoV was no longer detectable in plasma did not differ between patients in the hydrocort group and controls. The mean SARS-CoV RNA plasma level in the second and third week of illness were higher in the hydrocort than placebo group.	One patient was admitted to the ICU and eventually died; all other patients recovered and were discharged.	Risk of confounding. High-dose methylpred was given to 6 out of 7 patients in the placebo group, and 5 out of 10 patients in the hydrocort group.
Leung et al. (2005)	China	Prospective cohort study	8	4	Patients suspected of SARS, deceased, with autopsy findings consistent with ARDS	Hydrocort	Total dose 0.45-4.4g	NA	All patients treated with hydrocort had myofiber atrophy, whereas steroid-free patients did not. Focal myofiber necrosis was seen in 1 patient treated with steroids and 3 steroid-free patients.	Risk of confounding. SARS was not confirmed with serology or PCR. Critical illness myopathy cannot be distinguished from steroid myopathy in this study.
Wang et al. (2005)	Taiwan	Prospective cohort study	12	7	Hospitalized patients with SARS	1. Methyl-pred 2. Pred 3. Hydro-cort	Total daily dose 1. 1g or 1mg/kg 2. 20mg 3. 300mg	NA	At 60 days, patients that were treated with steroids had a high HRCT score, whereas all patients with a low HRCT score had not been treated with steroids.	Assessment of risk of confounding (by indication) is not possible (baseline data and disease severity are not provided). High loss-to-follow-up.
Xie et al. (2005)	China	Prospective cohort study	258	210	Patients recovered from (clinically diagnosed) SARS with at least two follow-up moments after discharge	UNK	Mean total dose (radiosone equivalent): 465.6 to 2447mg	NA	The percentage of patients receiving steroids, and the total dose of steroids were higher in patients with positive SARS-CoV IgG and a DLCO < 80% of predicted than for patients with negative SARS-CoV-IgG and patients with positive SARS-CoV-IgG with DLCO ≥ 80% of predicted.	Assessment of risk of confounding (by indication) is not possible (baseline data and disease severity are not provided). Diagnosis of SARS was not confirmed with PCR at the time of illness; 19.4% of included patients did not have SARS-CoV IgG.
Chen et al. (2006)	China	Retrospective cohort study	401	268	Patients with SARS	1. Methyl-pred 2. Hydro-pred 3. Dexa	Mean dose 131 mg/day, median total dose 1868 mg (type of steroid not stated)	NA	Mortality did not differ between steroid-treated and steroid-free patients. In patients with critical SARS (acute lung injury), steroid use was associated with reduced mortality (OR 0.083, 95% CI 0.007-0.956).	Risk of confounding (by indication). Given the small magnitude, the beneficial effect does not seem clinically relevant. Steroid use after 3 weeks was excluded.
Yam et al. (2007)	China	Retrospective cohort study	1287	1188	Hospitalized patients ≥ 18 years suspected of SARS treated with steroids <14 days of symptom onset or not treated with steroid	Hydrocort Methyl-pred Pred	UNK	NA	Mortality was lowest in patients treated with oral pred or low-dose methylpred. Mortality was highest in steroid-free patients and patients treated with high-dose methylpred. In univariate analysis, steroid treatment was associated with higher mortality. In a multivariate model, patients treated with low-dose methylpred showed a survival benefit over steroid-free patients.	Risk of confounding (by indication). Diagnosis of SARS was not confirmed with PCR or seroconversion.
Wei et al. (2009)	China	Retrospective cohort study	90	UNK	Hospitalized patients with SARS	Methyl-pred Hydrocort Dexa	UNK	NA	In univariate analysis, higher mean steroid dosage was associated with higher risk of death, but use of methylpred > 320 mg/day was not. In multivariate analysis, steroid use was not associated with a higher risk of mortality.	Assessment of risk of confounding (by indication) is not possible (baseline data and disease severity are not provided).

aHR, adjusted hazard ratio; aOR, adjusted odds ratio; CI, confidence interval; Dexa, dexamethasone; DLCO, diffusion capacity of the lung for carbon monoxide; HR, hazard ratio; Hydrocort, hydrocortisone ICU, intensive care unit; IQR, interquartile range; Methylpred, methylprednisolone; NA, not applicable; NNT, number needed to treat; OR, odds ratio; Pred, prednisone; RCT, randomized controlled trial; RECOVERY, Randomized Evaluation of COVID-19 therapy; UK, United Kingdom; UNK, unknown; USA, United Stated of America.

**Viral Load**

1. SARS-CoV-2: Five observational studies found that SARS-CoV-2 clearance time did not differ between steroid-treated patients and those not treated with steroids (Fang et al., 2020; Xu et al., 2020; Yuan et al., 2020; Zha et al., 2020; Zheng et al., 2020). Contrarily, three other observational studies report that steroid treatment was associated with a longer time until SARS-CoV-2 clearance (Chen et al., 2020; Ling et al., 2020; Xu et al., 2020). The risk of confounding was high in all these studies.
2. SARS-CoV: In a double-blinded RCT, patients with SARS were randomized to treatment with intravenous hydrocortisone (300 mg/day for 12 days) or placebo to study the effect of hydrocortisone on SARS-CoV clearance time (Lee et al., 2004). Patients with comorbidity, respiratory failure or symptoms for ≥ 5 days were excluded. In the second and third week of illness, the mean SARS-CoV RNA plasma levels were higher in the hydrocortisone group than in the placebo group, but the SARS-CoV clearance time did not differ between both groups. However, the risk of confounding is high, because high-dose methylprednisolone (500 mg/day) was given to 5 out of 10 patients in the hydrocortisone group and to 6 out of 7 patients in the placebo group. In a retrospective cohort study, steroid use was not associated with the viral load in lung tissue obtained at autopsy in 11 SARS patients (Mazzulli et al., 2004). However, the study may have been underpowered and the risk of confounding is high.
3. MERS-CoV: In an observational study in patients with MERS admitted to the ICU, steroid use was associated with a delay in MERS-CoV RNA clearance (Arabi et al., 2018). This study has a high risk of confounding.

**Clinical Outcome**

1. SARS-CoV-2: In an open-label RCT, patients with COVID-19 were randomized in a ratio of 1:2 to treatment with dexamethasone (6 mg daily, oral or intravenous, for a maximum of 10 days) at hospital admission or standard care (controls) (Recovery Collaborative Group et al., 2020). Age, sex, comorbidities and the requirement for respiratory support were similar in both groups. The median number of days since onset of symptoms was 8 days (IQR 5-13 days) for patients in the dexamethasone group and 9 days (IQR 5-13 days) for controls. Overall, the 28-day mortality rate was lower in dexamethasone-treated patients compared to controls (21.6 vs. 24.6%; age-adjusted RR 0.83, 95% CI 0.75–0.93). Similarly, dexamethasone use was associated with a lower 28-day mortality rate in the subgroups of patients requiring oxygen (RR 0.82, 95% CI 0.72–0.94) or invasive mechanical ventilation (RR 0.64, 95% CI 0.51–0.81) at randomization. Only in the subgroup of patients who were not treated with any type of respiratory support at randomization, there was no significant difference in the 28-day mortality rate between dexamethasone-treated patients and controls (RR 1.19, 95% CI 0.91–1.55). Dexamethasone use was also associated with a lower rate of invasive mechanical ventilation (RR 0.76, 95% CI 0.61–0.96) and shorter length of hospital stay (median 12 vs. 13 days).
  Six retrospective cohort studies also found that the use of steroids was associated with beneficial outcomes in patients with COVID-19 (Fadel et al., 2020; Fernandez Cruz et al., 2020; Lu et al., 2020; Sanz Herrero et al., 2020; Wang Y. et al., 2020; Wu et al., 2020). Two of these studies found that patients treated with steroids had a lower prevalence of mechanical ventilation and shorter length of ICU and hospital stay than controls (Fadel et al., 2020; Wang et al., 2020). In three other studies that only included COVID-19 patients with ARDS, the mortality rate was lower in patients treated with steroids than in controls (Fernandez Cruz et al., 2020; Lu et al., 2020; Wu et al., 2020). In COVID-19 patients treated with tocilizumab, additional administration of steroids was also associated with a lower mortality rate (Sanz Herrero et al., 2020).
  In contrast, two retrospective cohort studies found that steroid use was associated with detrimental outcome (Giacobbe et al., 2020; Mo et al., 2020). The first study included COVID-19 patients who were admitted to the ICU. They found that methylprednisolone use was independently associated with development of a blood stream infection (Giacobbe et al., 2020). Another retrospective cohort study found that patients treated with steroids were less likely to reach clinical and radiological remission within 10 days than patients who did not use steroids (Mo et al., 2020). However, this study has a high risk of confounding by indication.
  Lastly, one cohort study in hospitalized COVID-19 patients found that the rate of steroid treatment was not significantly different between surviving and deceased patients (Cao et al., 2020). Again, there was significant risk of confounding.
2. SARS-CoV: Similar to the results for SARS-CoV-2, observational studies in patients with SARS have different results. In one cohort study that included patients with severe SARS, steroid use was associated with reduced mortality (Chen et al., 2006). Three other cohort studies found that treatment with steroids had no effect on the mortality rate (Chen et al., 2006; Wei et al., 2009) or development of ARDS (Chen et al., 2005) in patients with SARS.
  Several other cohort studies report that steroid use was associated with detrimental outcome (Auyeung et al., 2005; Leung et al., 2005; Ng et al., 2004; Wang et al., 2005; Xie et al., 2005; Yam et al., 2007). Two retrospective cohort studies report that steroid use was associated with a higher risk of ICU admission and mortality (Auyeung et al., 2005; Yam et al., 2007). In two studies that included patients who recovered from SARS, steroid use was associated with the presence of CT abnormalities and a lower diffusion capacity of the lungs during follow-up (Ng et al., 2004; Wang et al., 2005; Xie et al., 2005). However, the risk of confounding by indication is high. Another cohort study investigated autopsy findings of 8 SARS patients with ARDS. They found that all patients with hydrocortisone had myofiber atrophy, whereas this was absent in patients who were not treated with steroids (Leung et al., 2005). However, critical illness neuropathy could not be distinguished from steroid myopathy in this study.
3. MERS-CoV: One retrospective cohort study included MERS patients who were admitted to the ICU. Steroid use was associated with a higher 90-day mortality in a multivariable logistic regression model. However, in a Cox proportional hazard model, steroid use was not associated with a higher mortality rate (Arabi et al., 2018). This suggests that their statistical models did not adjust for all confounders. Another retrospective cohort study found that steroid use was associated with a higher mortality in hospitalized MERS patients (Alfaraj et al., 2019). It was not possible to assess the risk of bias in this study, because essential information is missing, such as baseline characteristics.

**Effect of Steroid Dose**

1. SARS-CoV-2: Two retrospective cohort studies found that a higher steroid dose was associated with death and prolonged time until viral clearance (Li S. et al., 2020), while use of low-dose steroids was not (Li S. et al., 2020; Li X. et al., 2020). Another retrospective cohort study found that each 10 mg increase in steroid dose was associated with a 4% increase in the risk of mortality after 28 days in COVID-19 patients with ARDS or sepsis (Lu et al., 2020). However, the steroid dose tended to be the highest in the patients who were most severely ill. Thus, there is significant risk of confounding by indication in these studies.
2. SARS-CoV: In one retrospective cohort study, a lower mean steroid dose was associated with a higher 28-day mortality and mechanical ventilation rate (Gomersall et al., 2004). Contrarily, another retrospective cohort study found that a higher mean steroid dose was associated with a higher mortality rate (Wei et al., 2009). Two other retrospective cohort studies found no association between steroid dose and clinical outcome (Ho et al., 2003; Hui et al., 2005). The first study found that the rate of ICU admission, mechanical ventilation or mortality after 3 weeks were not significantly different for patients treated with high-dose or low-dose steroids (Ho et al., 2003). The other study reports that steroid dose was not associated with the results of the 6-minute walking test or the severity of radiographic abnormalities at 6 months after SARS (Hui et al., 2005). In all of these studies, the risk of confounding (by indication) is high.
3. MERS-CoV: No studies available.

**Timing of Steroid Administration**

1. SARS-CoV-2: No studies available.
2. SARS-CoV: Two observational studies investigated whether the timing of steroid administration was associated with clinical outcomes in patients with SARS (Zhao et al., 2003; Jang et al., 2004). The first study reports that the time between onset of symptoms and start of steroids was not associated with the risk of ICU admission or mechanical ventilation (Jang et al., 2004). The second study found that early treatment with high-dose methylprednisolone might be beneficial for SARS patients (Zhao et al., 2003). In this study, treatment with methylprednisolone was started at different moments in the course of the disease: (A) if patients had not recovered after 14 days, (B) if symptoms or radiological abnormalities worsened, or (C) if fever persisted for ≥ 3 days after admission. The rate of mechanical ventilation was 7, 33, and 0%, respectively, and the mortality rate was 6, 12, and 0%, respectively. However, patients were not randomized to different treatment protocols, and consequently, baseline characteristics and disease severity were different. In both studies, the risk of confounding is high.
3. MERS-CoV: No studies available.

### Calcineurin Inhibitors (CNI): Cyclosporin A (CsA) and Tacrolimus (TAC)

CNIs (cyclosporine A (CsA) and tacrolimus (TAC)) are used to prevent rejection after organ transplantation and to treat autoimmune diseases, like inflammatory bowel disease. CsA and TAC inhibit T-cell activation. *In-vivo*, CsA and TAC form complexes with cyclophilins and FK506-binding proteins, respectively. These complexes prevent the phosphatase activity of calcineurin. As a result, the dephosphorylation of the nuclear factor of activated T cells is decreased (Carbajo-Lozoya et al., 2012; Ma et al., 2016).

In addition, cyclophilins, the binding proteins of CsA, catalyze the cis/trans isomerization of propyl peptide bonds. This is an essential step in correct folding of proteins, such as cellular and viral proteins (Ma-Lauer et al., 2020). This function of cyclophilin A is found to be essential for the replication of SARS-CoV-2 and other viruses belonging to the Nidovirales order (Carbajo-Lozoya et al., 2014).

**Viral Load**

1. SARS-CoV-2: No studies available.
2. SARS-CoV and MERS-CoV: Several *in-vitro* studies showed that CsA significantly inhibits the viral replication and the cytopathic effect (CPE: the virus-induced changes in host cells that cause cell death) of SARS-CoV and MERS-CoV in infected cells (Vero, Huh7, Calu-3, and human lung tissue) in a dose-dependent manner (de Wilde et al., 2011; Pfefferle et al., 2011; Carbajo-Lozoya et al., 2012; de Wilde et al., 2013; Li et al., 2018; Sauerhering et al., 2020). One of these studies found that a high concentration of CsA (15 µM) completely inhibited the CPE, without affecting the viability of the cells (de Wilde et al., 2013). Next to these *in-vitro* effects, CsA also inhibited MERS-CoV viral replication and reduced cellular apoptosis in *ex-vivo* cultures of bronchial and lung tissue (Li et al., 2018).

Similar to CsA, TAC inhibited the viral replication of SARS-CoV in Vero cells in a dose-dependent manner (Carbajo-Lozoya et al., 2012). In this study, high-dose TAC reduced SARS-CoV titers 11.112-fold after only 24 h (Carbajo-Lozoya et al., 2012).

**Clinical Outcome**

No studies matching the inclusion criteria.

### Antimetabolites

#### Mycophenolic Acid (MPA)

Mycophenolic acid (MPA) and its prodrugs, mycophenolate mofetil (MMF) and mycophenolate sodium, are used in the treatment of autoimmune diseases and to prevent rejection in organ transplant recipients. MPA inhibits inosine-5’-monophosphate dehydrogenase, which leads to depletion of intracellular guanosine and deoxyguanosine nucleotides. This suppresses DNA synthesis and thus proliferation of T and B lymphocytes (Villarroel et al., 2009).

**Viral Load**

1. SARS-CoV-2: One *in-vitro* study found that MPA inhibits SARS-CoV-2 replication in VeroE6/TMPRSS2 cells (Kato et al., 2020). In another study (Han et al., 2020), human pluripotent stem cells (hPSC) were differentiated into lung organoids and then infected with SARS-CoV-2. In these lung organoids, MPA inhibited viral replication while the CPE of SARS-CoV-2 was still observed, even with high concentrations of MPA.
2. SARS-CoV: MPA does not inhibit the proteolytic activity of SARS-CoV PL^pro^ (Cheng et al., 2015) or SARS-CoV replication in Vero cells (Barnard et al., 2006).
3. MERS-CoV: Two studies showed that MPA effectively inhibits the proteolytic activity of the papain-like protease (PL^pro^) of MERS-CoV (Cheng et al., 2015; Lin et al., 2018). PL^pro^ is responsible for the cleavage of nonstructural proteins, which are essential for viral maturation. Three other *in-vitro* studies showed that MPA significantly inhibited the replication and CPE of MERS-CoV in Vero cells (Chan et al., 2013; Hart et al., 2014; Shen et al., 2019). This effect was dose-dependent.

In contrast, an *in-vivo* study in marmosets infected with MERS-CoV found that the mean viral load in the lungs was higher in MMF-treated animals than in controls (Chan et al., 2015). However, since MERS-CoV does not cause lethal disease in marmosets, this animal model does not adequately resemble human MERS (Johnson et al., 2015).

**Clinical Outcome**

1. SARS-CoV-2: No studies available.
2. SARS-CoV: No studies available.
3. MERS-CoV: In a retrospective cohort study of 51 hospitalized patients with MERS, eight patients (16%) had received MMF as experimental treatment for MERS. Overall, 19 (37%) patients were admitted to the ICU and eventually died. All other patients survived. In univariate analysis, MMF treatment was associated with survival. However, MMF was given to the less severely ill patients and, seven of the eight MMF-treated patients (87.5%) were also treated with interferon beta (Al Ghamdi et al., 2016). Thus, there is significant risk of confounding.

#### Thiopurine Analogues

Thiopurine analogues (azathioprine (AZA) and 6-mercaptopurine (6MP)) are used as anticancer treatment, to prevent rejection in organ transplant recipients and as treatment of several chronic autoimmune diseases. AZA is a prodrug of 6MP. *In-vivo*, 6MP is converted into 6-thioguanine (6TG) which is incorporated into cellular DNA. This prevents further DNA replication (Chen et al., 2009).

**Viral Load**

1. SARS-CoV-2: No studies available.
2. SARS-CoV and MERS-CoV: 6MP and 6TG effectively inhibit the proteolytic activity of PL^pro^ of MERS-CoV and SARS-CoV in a dose-dependent manner (IC_50_ of 12 to 27 µM) in inhibition assays with peptide and fluorogenic substrates (Chou et al., 2008; Cheng et al., 2015; Lin et al., 2018). These results suggest that 6MP and 6TG can inhibit the replication of MERS-CoV and SARS-CoV *in-vitro*.

**Clinical Outcome**

No studies matching the inclusion criteria.

### Mammalian Target of Rapamycin (mTOR) Inhibitors

Sirolimus (rapamycin) and everolimus are mTOR inhibitors. These drugs are used to prevent acute rejection in organ transplantation (Pfefferle et al., 2011). In higher doses, everolimus is also used as anticancer drug (Pfefferle et al., 2011). *In-vivo*, sirolimus and everolimus bind to the FK-binding protein 12 (FKBP-12). This sirolimus/everolimus-FKBP-12 complex binds to mTOR, a phosphatidylinositol kinase-related kinase. This inhibits protein synthesis, cell cycle progression and cell growth (Wullschleger et al., 2006).

**Viral Load**

1. SARS-CoV-2: No studies available.
2. SARS-CoV: No studies available.
3. MERS-CoV: One *in-vitro* study showed that sirolimus and everolimus dose-dependently reduce MERS-CoV infection in a hepatocyte derived cell line. For both drugs, the cytotoxicity was < 10% (Kindrachuk et al., 2015).

**Clinical Outcome**

No studies matching the inclusion criteria.

### Anti-Cytokine Agents

#### Anti-Tumor-Necrosis-Factor-α (TNF- α) Agents

There are several types of anti-tumor-necrosis-factor-α (TNF-α) agents: infliximab, adalimumab and golimumab (monoclonal antibodies against TNF-α), certolizumab (TNF-α binding fragment of a monoclonal antibody) and etanercept (fusion protein composed of the extracellular portion of the TNF-receptor-2 and the Fc portion of immunoglobulin G1). Anti-TNF- α agents are used in the treatment of autoimmune diseases, like rheumatoid arthritis and inflammatory bowel disease. TNF-α is a pro-inflammatory cytokine that recruits neutrophils and monocytes to the area of inflammation and activates intracellular signaling in various cells of the immune system (Mitoma et al., 2018).

**Viral Replication**

No studies matching the inclusion criteria.

**Clinical Outcome**

1. SARS-CoV-2: No studies available.
2. SARS-CoV: In an animal study, mice infected with SARS-CoV were treated with an anti-TNF-α monoclonal antibody or an isotype-matched control antibody. In mice treated with the anti-TNF-α monoclonal antibody, the onset of weight loss and respiratory illness was delayed compared to controls. However, the mortality rate after 10 days was similar in both groups (Nagata et al., 2008).
3. MERS-CoV: No studies available.

#### Anakinra

Anakinra is an IL-1 receptor antagonist that is registered for the treatment of several autoinflammatory diseases, such as adult-onset Stills disease and familial Mediterranean fever (Cavalli et al., 2020).

**Viral Replication**

No studies matching the inclusion criteria.

**Clinical Outcome**

1. SARS-CoV-2: A retrospective cohort study (Cavalli et al., 2020) included patients with COVID-19 with moderate-to-severe ARDS and hyperinflammation (CRP ≥ 100 mg/L and/or ferritin ≥ 900 ng/mL). Patients treated with mechanical ventilation or other anti-inflammatory agents were excluded. Patients in the intervention group (n = 29) received high-dose anakinra (5mg/kg twice daily intravenously). The control group (n = 16) consisted of patients who retrospectively met the eligibility criteria for anakinra, but who presented to the hospital before the availability of the drug. The 3-week mortality rate was lower in anakinra-treated patients compared to controls (HR = 0.20, 95% CI 0.04–0.63, p = 0.009), but more anakinra-treated patients than controls still required mechanical ventilation after 3 weeks (17 vs. 6%). The percentage of patients that was discharged and had resumed normal activities after 3 weeks was not significantly different (45 vs. 44%). A limitation of this study is the significant risk of confounding. For example, there were significant differences between patients treated with anakinra and controls.
2. SARS-CoV: No studies available.
3. MERS-CoV: No studies available.

#### Tocilizumab and other IL-6 Inhibitors

IL-6 is an important pro-inflammatory cytokine that is involved in the acute phase response and differentiation and function of B and T cells (Mosharmovahed et al., 2020). Of the three commercially available IL-6 inhibitors (tocilizumab, sarilumab and siltuximab), tocilizumab is the most well-known (Khiali et al., 2020). Tocilizumab is a recombinant humanized anti-IL-6 receptor monoclonal antibody. Tocilizumab is used to treat several autoinflammatory diseases, like rheumatoid arthritis, giant cell arteritis and the chimeric antigen receptor T-cell induced cytokine storm syndrome (Khiali et al., 2020). Notably, Il-6 inhibitors were not available at the time of the SARS and MERS pandemics. The nine retrospective cohort studies matching the inclusion criteria are summarized in **Table 3**.

**Table 3:** Retrospective cohort studies: IL-6 inhibitors in the treatment of patients with COVID-19.

Study	Subjects receiving IL-6 inhibitor	Type and dosage IL-6 inhibitor	Controls (n)	Main conclusion	Risk of bias
Harmful					
Quartuccio et al. (2020)	42 patients with severe COVID-19	Tocilizumab 8 mg/kg	69	Mortality rate (after a follow-up of 1 to 2 months), 16.7% in tocilizumab group 16.7% vs. 0% in controls. Bacterial infection: 42.9% in tocilizumab group 42.9% vs. 0% in controls.	Risk of confounding: high (tocilizumab-treated patients were more severely ill, significantly older and more frequently received antivirals and glucocorticoids).
No effect					
Campochiaro et al. (2020)	32 patients with severe COVID-19 pneumonia and CRP ≥ 100 mg/L or ferritin ≥ 900 ng/mL	Tocilizumab 400 mg once or twice	33	1-month mortality rate: not significantly different (16% of tocilizumab group vs. 33% of controls, p = 0.150). Discharge rate, duration of hospitalization and serious adverse event rate (including infections and bacteremia) also not significantly different.	Risk of confounding: low (patient characteristics and disease severity at admission not significantly different. Eligibility criteria for tocilizumab clearly defined. Controls recruited among patients who fulfilled eligibility criteria before and after time period of tocilizumab availability).
Colaneri et al. (2020)	21 patients with COVID-19, and CRP > 5 mg/dl, procalcitonin < 0.5 ng/ml, paO2/fiO2 ratio < 300 and ALT < 500 U/l	Tocilizumab, dose UNK	91 (propensity-score matched)	In a logistic regression model, the use of tocilizumab was not significantly associated with mortality (OR 0.78, 95% CI 0.06 – 9.34) or ICU admission (OR 0.11, 95% CI 0.00 – 3.38).	Risk of confounding: moderate. All patients were treated with methylprednisolone 1 mg/kg for 10 days.
Giacobbe et al. (2020)	23 patients with COVID-19 admitted to the ICU for > 48 h	Tocilizumab, dose UNK	55	In multivariate analysis, tocilizumab use was not significantly associated with blood stream infection (HR 1.21, 95% CI 0.44 – 3.30).	Risk of confounding: some patients were also treated with methylprednisolone (which was independently associated with a higher incidence of blood stream infection). Complete assessment of risk of bias is not possible, as baseline characteristics of the tocilizumab-treated patients vs. controls are lacking.
Rojas-Marte et al. (2020)	96 patients with severe COVID-19	Tocilizumab, dose UNK	97 (matched by oxygen requirement)	The mortality rate was not significantly different between both groups (52% s. 62%, p = 0.09), and neither was the length of hospital stay (15 vs. 17 days, p = 0.32). When excluding intubated patients, the mortality was lower in the tocilizumab group compared to controls (6% vs. 27%, p = 0.024).	Risk of confounding: high (concomitant use of other drugs; the controls are older and more comorbidities (e.g. diabetes, heart failure); significantly more controls than tocilizumab-treated patients with bacteremia (23.7 vs. 12.5%, p=0.04). Selection bias: patients who died within 24 h of admission and patients included in clinical trials with other biological agents than tocilizumab were excluded.
Beneficial					
Capra et al. (2020)	62 patients with severe COVID-19	Tocilizumab 324, 400 or 800 mg	23	3-week survival rate (adjusted for age, comorbidities and SARS-CoV-2 viral load at admission): significantly higher in tocilizumab-treated patients than in controls (HR for death 0.035, 95% CI 0.004 – 0.347). There were no tocilizumab-related infections.	Risk of confounding: low (primary outcome is adjusted for important differences between both groups). Follow-up: the 3-week follow-up was completed in only 4 out of the 85 patients.
Della-Torre et al. (2020)	28 patients with severe COVID-19, bilateral pneumonia and hyperinflammation	Sarilumab 400 mg	28 (matched for age, sex, comorbidity, inflammatory markers, respiratory parameters, CT findings)	28-day mortality: not significantly different (HR 0.36, 95% CI 0.08 – 1.68) Time to death: longer in sarilumab group than in controls (19 vs. 4 days, p = 0.006). Mechanical ventilation rate: not significantly different (21 vs. 25%, p = 0.99). Clinical improvement after 28 days: not significantly different (sarilumab group 61% vs. controls 64%). Time to clinical improvement: shorter in sarilumab-treated patients (10 days vs. 24 days, p = 0.01). Infection rate: not significantly different (21% vs. 18% of patients, p = 0.99).	Risk of confounding: moderate (characteristics of patients and controls not significantly different; but there is risk of confounding by indication, because it is unclear how it was decided who would receive sarilumab and who would not).
Klopfenstein et al. (2020)	20 patients with severe COVID-19, failure of standard care	Tocilizumab, dose UNK	25	Mechanical ventilation: lower in tocilizumab-treated patients than in controls (0% vs. 32%, p = 0.006). Mortality: not significantly different (25% tocilizumab-treated patients vs. 48% controls, p = 0.066). Death and/or ICU admission rate (combined endpoint): lower in tocilizumab-treated patients than in controls (25% vs. 72%, p = 0.002).	Risk of confounding: moderate (the most critically ill patients were selected to receive tocilizumab. Higher CRP and oxygen requirement, more patients > 70 years old and higher CCI in tocilizumab-treated patients than in controls; however, in case of bias this would result in a more detrimental outcome in tocilizumab-treated patients (which was not observed)). Selection bias: patients treated with remdesivir or immunoglobulins were excluded.
Moreno-García et al. (2020) (preprint, no peer-review)	77 patients with COVID-19, not admitted to ICU within 24h after admission	Tocilizumab 1-3 doses of 400 mg (≤ 75 kg) or 800 mg (> 75kg)	94	ICU admission rate: lower in tocilizumab-treated patients than in controls (10.3% vs. 27.6%, P= 0.005). Mechanical ventilation rate: lower in tocilizumab-treated patients (0 vs 13.8%, P=0.001). In multivariate analysis, tocilizumab use was significantly associated with better outcome (OR for ICU admission and/or death 0.03, 95% CI 0.007 – 0.1).	Risk of confounding: moderate (tocilizumab-treated patients were more severely ill, had higher CRP and were more frequently treated with steroids than controls; however, in case of bias this would result in a more detrimental outcome in tocilizumab-treated patients (which was not observed)).

ALT, alanine aminotransferase; CCI, Charlson comorbidity index; CI, confidence interval; CRP, C-reactive protein; CT, computer tomography; fiO2, fractional inspired oxygen; GI, gastro-intestinal; HR, hazard ratio; ICU, intensive care unit; IQR, interquartile range; NA, not applicable; OR, odds ratio; paO2, arterial partial pressure of oxygen; PCR, polymerase chain reaction; UNK, unknown.

**Viral Replication**

No studies matching the inclusion criteria.

**Clinical Outcome**

1. SARS-Cov-2: The nine studies matching the inclusion criteria report conflicting results. One retrospective cohort study found that treatment of COVID-19 patients with tocilizumab was associated with a higher mortality rate and higher prevalence of bacterial infection (Quartuccio et al., 2020). However, tocilizumab-treated patients were more severely ill than controls. They were also significantly older and more frequently received antivirals and glucocorticoids than controls.
  In four other studies, the mortality and ICU admission rate were not significantly different between patients treated with an IL-6-inhibitor (tocilizumab or sarilumab) and controls (Campochiaro et al., 2020; Colaneri et al., 2020; Della-Torre et al., 2020; Rojas-Marte et al., 2020). Similarly, the discharge rate (Campochiaro et al., 2020), duration of hospitalization (Campochiaro et al., 2020), blood stream infections (Giacobbe et al., 2020), serious adverse event rate (including infections and bacteremia) (Campochiaro et al., 2020) and rate of clinical improvement after 28 days (Della-Torre et al., 2020) were not significantly different. In three of these studies (Colaneri et al., 2020; Della-Torre et al., 2020; Rojas-Marte et al., 2020), the controls were matched to the tocilizumab-treated patients on several important patient characteristics in order to reduce the risk of confounding by indication. In the fourth study (Campochiaro et al., 2020), the patient characteristics and disease severity at admission were not significantly different between both groups and the eligibility criteria for tocilizumab were clearly defined. Moreover, controls were recruited among patients who fulfilled these eligibility criteria before and after the time period of tocilizumab availability.
  Lastly, three other studies report a higher 3-week survival rate (Capra et al., 2020), a lower rate of mechanical ventilation (Klopfenstein et al., 2020) and a lower rate of ICU admission (Moreno-García et al., 2020) in patients treated with tocilizumab. Remarkably, in two of these studies, the tocilizumab-treated patients were more critically ill at admission than controls, but their outcome was better.
  In summary, one study found that tocilizumab was associated with a higher mortality rate, but this study has a high risk of confounding. Four observational studies found no effect of tocilizumab or sarilumab and four other studies showed a beneficial effect of tocilizumab.
2. SARS-CoV: No studies available.
3. MERS-CoV: No studies available.

### Immunosuppressive Drugs Without Studies Matching Eligibility Criteria

We could not identify any studies matching our inclusion criteria for the following drugs: abatacept, alemtuzumab, basiliximab, belatacept, brodalumab, cyclophosphamide, dupilumab, eculizumab, ixekizumab, methotrexate, rituximab, and secukinumab.

## Discussion

There are 30 studies meeting the inclusion criteria for our review that studied the effect of immunosuppressive drugs on infection with SARS-CoV-2. These studies investigated the effects of corticosteroids, mycophenolic acid (MPA), anakinra or tocilizumab.

Interestingly, MPA inhibits SARS-CoV-2 replication *in-vitro* (Han et al., 2020; Kato et al., 2020). This is in contrast with COVID-19 guidelines aimed at patients using immunosuppressive drugs for a pre-existing disease (Lopez et al., 2020; British Transplantation Society, 2020; Société Francophone de Transplantation (SFT) SFdN et al., 2020). These expert-opinion-based guidelines recommend to discontinue MPA or reduce the MPA dose in patients with COVID-19. Based on the results of our review, this approach can be questioned. Clinical studies are needed to confirm the inhibitory effect of MPA on SARS-CoV-2 replication *in-vivo*.

The results of some clinical studies suggest that corticosteroids are beneficial in patients with COVID-19, especially in mitigating the effects of the cytokine storm. An RCT found that dexamethasone use was associated with a lower 28-day mortality rate, a shorter length of hospital stay and a lower prevalence of mechanical ventilation (Recovery Collaborative Group et al., 2020). The beneficial effect of dexamethasone was greatest in the most severely ill patients. For example, in patients treated with mechanical ventilation, dexamethasone reduced the risk of mortality with 36% (95% CI 19–49%), whereas dexamethasone had no effect on the mortality rate of patients who did not require oxygen support. The results of this RCT support our hypothesis that immunosuppressive drugs can be used to prevent and treat the hyperinflammatory phase of COVID-19.

Six cohort studies confirmed that steroid use was associated with a better clinical outcome in patients with COVID-19 (Fadel et al., 2020; Fernandez Cruz et al., 2020; Lu et al., 2020; Sanz Herrero et al., 2020; Wang et al., 2020; Wu et al., 2020). However, two cohort studies have opposite results (Giacobbe et al., 2020; Mo et al., 2020). They found that steroid use was associated with a higher risk of blood stream infections (Giacobbe et al., 2020) and a lower 10-day remission rate (Mo et al., 2020). Unfortunately, all cohort studies have a high risk of confounding (by indication), which limits their validity and generalizability. Thus, more high-quality studies are needed to confirm the effect of steroids on the clinical outcome of COVID-19.

Moreover, it has to be established whether corticosteroids have an effect on the host immune response against the virus. An adequate response of the immune system in the first phase of COVID-19 can prevent progression to viral pneumonia (Shi et al., 2020). As expected, there were no studies that found that corticosteroid use was associated with accelerated SARS-CoV-2 clearance. However, some cohort studies found that steroid use was associated with a delay in SARS-CoV-2 clearance (Chen et al., 2020; Ling et al., 2020; Xu et al., 2020), while other studies report that steroid use was not associated with SARS-CoV-2 clearance time (Fang et al., 2020; Xu et al., 2020; Yuan et al., 2020; Zha et al., 2020; Zheng et al., 2020). These studies have a high risk of confounding (by indication). Thus, we could not draw firm conclusions on the effect of corticosteroids on viral replication.

There is growing interest in IL-6 inhibitors, like tocilizumab. Patients with severe COVID-19 have high levels of IL-6 (Mosharmovahed et al., 2020) and in three cohort studies, tocilizumab use was associated with lower mortality and ICU admission rate (Capra et al., 2020; Klopfenstein et al., 2020; Moreno-García et al., 2020). However, other cohort studies report contradictory results (Campochiaro et al., 2020; Colaneri et al., 2020; Della-Torre et al., 2020; Giacobbe et al., 2020; Quartuccio et al., 2020; Rojas-Marte et al., 2020). Furthermore, the risk of confounding in these observational studies is high. As a result, it is not clear yet whether IL-6 inhibitors can be used to prevent and treat the cytokine storm. Fortunately, several research groups have published a protocol for a RCT to assess the efficacy and safety of tocilizumab as treatment of COVID-19 (Cellina et al., 2020; Fu et al., 2020; Rilinger et al., 2020).

The observational studies for corticosteroids and IL-6 inhibitors report contradictory results, which is probably related to heterogeneity in study populations. In addition, there could have been differences in local treatment protocols. These factors limit the possibility of conducting a meta-analysis. Furthermore, in many studies there are differences in patient characteristics (such as age, gender and comorbidity) and treatment regimens between patients in the intervention group and control group. Moreover, in many cases, the most severely ill patients were treated with immunosuppressive drugs, whereas less severely ill patients were not. This might have resulted in confounding by indication.

Currently, the number of studies on the effect of immunosuppressive drugs on COVID-19 is still limited. SARS and MERS are also caused by a coronavirus and share many features with COVID-19 (Wang D. et al., 2004; Yin and Wunderink, 2018; Wang D. et al., 2020). Thus, the effects of immunosuppressive drugs on these viruses may resemble the effects on SARS-CoV-2. We were able to include 39 additional studies that studied the effect of immunosuppressive drugs on infection with SARS-CoV and MERS-CoV. CNIs and mTOR inhibitors inhibit the viral replication of SARS-CoV and MERS-CoV *in-vitro*. There is also indirect evidence that thiopurine analogues inhibit the viral replication of SARS-CoV. It would therefore be interesting to investigate the effect of CNIs, mTOR inhibitors and thiopurine analogues on the viral replication of COVID-19.

Notably, there are some important differences between SARS-CoV-2, SARS-CoV and MERS-CoV. First, the nucleotide sequence of the genome of SARS-CoV-2 is only 79.7% similar to SARS-CoV (Zhou et al., 2020). However, the similarity of the nucleotide sequence of envelope, nucleocapsid and spike proteins is as high as 96, 90, and 77%, respectively (Zhou et al., 2020). The similarity of the genome of SARS-CoV-2 and MERS-CoV is only 54%, and for the spike protein, a transmembrane protein involved in binding of the virus to the host receptor, 31.9% (Zhou et al., 2020). Second, MERS-CoV uses dipeptidyl peptidase 4 (DPP4) as host cell receptor (Raj et al., 2013), while SARS-CoV-2 and SARS-CoV use ACE2 (Li et al., 2003; Hoffmann et al., 2020). Lastly, the case fatality rate of MERS-CoV is much higher than of COVID-19 and SARS. While interpreting the results of this review, these differences should be kept in mind.

Our study has some limitations. First, we did not fully meet all the requirements of a systematic review, since we have only searched one database and there was no second reviewer. Second, we only included “genuine” immunosuppressive drugs in our review. As a consequence, we excluded antiviral/antiparasitic drugs, like (hydroxy)chloroquine, despite the fact that some would argue that this drug has immunosuppressive properties.

## Conclusions

Some immunosuppressive drugs may be beneficial in the treatment of COVID-19. MPA inhibits SARS-CoV-2 replication *in-vitro*. There are indications that corticosteroids and IL-6 inhibitors, like tocilizumab, can reduce mortality and prevent mechanical ventilation in patients with COVID-19. These results have to be confirmed in high-quality clinical trials before these drugs can be implemented as standard care. Based on the positive results of CNIs, mTOR inhibitors and thiopurine analogues in *in-vitro* studies with SARS-CoV and MERS-CoV, it would be interesting to investigate their effects on SARS-CoV-2 replication.

## Author Contributions

RM: idea for the review. All authors: research design. TS: PubMed search, data extraction, writing first draft of the manuscript. All authors contributed to the article and approved the submitted version.

## Conflict of Interest

The authors declare that the research was conducted in the absence of any commercial or financial relationships that could be construed as a potential conflict of interest.
