# Supplementary material for: Immunosuppressive Drugs and COVID-19: A Review
Source: Front Pharmacol. 2020 Aug 28;11:1333. doi: 10.3389/fphar.2020.01333 (PMC7485413; doi:10.3389/fphar.2020.01333)
Supplement: Supplementary file 1 [file DataSheet_1.pdf]

Supplementary file 1 – PubMed search

"COVID-19 drug treatment" [Supplementaary Concept] OR "COVID-19" [Supplementary Concept] OR "Middle East Respiratory Syndrome Coronavirus"[Mesh] OR "SARS Virus"[Mesh] OR "Coronavirus"[Mesh] or coronavirus* [tiab] or covid* [tiab] or ncov* [tiab] or sars* [tiab] or mers* [tiab]

AND

- "Mycophenolic Acid"[Mesh] or mycophenol* [tiab] or cellcept [tiab] or myfortic [tiab] → 41 results
- "Cyclosporine"[Mesh] OR cyclospor* [tiab] OR ciclospor* [tiab] OR neoral [tiab] OR cya-NOF [tiab] OR sandimmune [tiab] OR csa-neoral [tiab] OR csa neoral [tiab] → 71 results
- "Tacrolimus"[Mesh] OR tacrolimus [tiab] OR prograf* [tiab] → 26 results
- "Thioguanine"[Mesh] OR thioguanin* [tiab] OR tioguanin* [tiab] OR 6-thioguanin* [tiab] OR 6-tioguanin* [tiab] OR lanvis [tiab] OR purinethiol* [tiab] OR 6-purinethiol* [tiab] OR "Mercaptopurine"[Mesh] OR mercaptopurin* [tiab] OR 6-mercaptopurin* [tiab] OR thiopurin* [tiab] OR purinethol [tiab] OR puri-nethol [tiab] OR "Azathioprine"[Mesh] OR azathioprine [tiab] OR imurel [tiab] OR imuran [tiab] OR immuran [tiab] → 23 results
- "Methotrexate"[Mesh] OR methotrexate* [tiab] → 30 results
- "Sirolimus"[Mesh] OR sirolimus [tiab] OR rapamycin [tiab] OR rapamune [tiab] OR "Everolimus"[Mesh] OR everolimus [tiab] OR afinitor [tiab] OR certican [tiab] OR mtor [tiab] → 53 results
- "Glucocorticoids"[Mesh] or corticoid* [tiab] or corticoster* [tiab] or steroid* [tiab] or glucocorticoid* [tiab] or "Prednisone"[Mesh] or predniso* [tiab] or cortisol* [tiab] or "Hydrocortisone"[Mesh] or hydrocortiso* [tiab] or methylpredniso* [tiab] or dexamethaso* [tiab] → 586 results
- "Cyclophosphamide"[Mesh] OR cyclophosphamide [tiab] OR cytophosphan* [tiab] OR sendoxan [tiab] OR cytoxan [tiab] OR endoxan [tiab] OR neosar [tiab] OR procytox [tiab] OR cyclophosphan* [tiab] → 39 results
- "Rituximab"[Mesh] OR rituximab [tiab] OR CD20* [tiab] OR CD-20* [tiab] OR anti-CD20* [tiab] OR anti-CD-20* [tiab] OR mabthera [tiab] OR rituxan [tiab] → 57 results
- "thymoglobulin" [Supplementary Concept] OR anti-thymocyte* [tiab] OR thymocyte* [tiab] OR antithymocyte [tiab] OR ATG [tiab] OR rATG [tiab] OR r-ATG [tiab] OR "Alemtuzumab"[Mesh] OR alemtuzumab [tiab] OR campath* [tiab] OR lemtrada [tiab] → 41 results
- "tocilizumab" [Supplementary Concept] OR tocilizumab [tiab] OR atlizumab [tiab] OR Actemra [tiab] OR roactemra [tiab] → 226 results

- "Basiliximab"[Mesh] OR basiliximab [tiab] OR simulect [tiab] → 0 results
- "Interleukin 1 Receptor Antagonist Protein"[Mesh] OR interleukin 1 receptor antagonist*[tiab] OR IL1 receptor antagonist* [tiab] OR IL1 inhibitor* [tiab] OR interleukin 1 inhibitor* [tiab] OR interleukin-1 receptor antagonist* [tiab] OR IL-1 receptor antagonist* [tiab] OR IL-1 inhibitor* [tiab] OR interleukin-1 inhibitor* [tiab] OR anakinra [tiab] OR antril [tiab] OR kineret [tiab] → 29 results
- "dupilumab" [Supplementary Concept] OR dupilumab [tiab] → 3 results
- "brodalumab" [Supplementary Concept] OR brodalumab [tiab] OR siliq [tiab] → 0 results
- "secukinumab" [Supplementary Concept] OR Secukinumab [tiab] OR cosentyx [tiab] → 0 results
- "ixekizumab" [Supplementary Concept] OR Ixekizumab [tiab] OR taltz [tiab] → 1 result
- "Infliximab"[Mesh] OR infliximab* [tiab] OR remicade [tiab] OR inflectra [tiab] OR renflexis [tiab] OR anti-TNF* [tiab] OR anti TNF* [tiab] OR anti-tumor* [tiab] OR anti-tumour* [tiab] OR anti tumor* [tiab] OR anti tumour* [tiab] OR "Adalimumab"[Mesh] OR adalimumab* [tiab] OR humira [tiab] OR amjevita [tiab] OR cyltezo [tiab] OR "Etanercept"[Mesh] OR etanercept [tiab] OR TNFR-Fc [tiab] OR TNFR Fc [tiab] OR TNTR Fc [tiab] OR Erelzi [tiab] OR enbrel [tiab] OR TNF receptor type II-IgG [tiab] OR TNF-receptor type II-IgG [tiab] OR "Certolizumab Pegol"[Mesh] OR certolizumab [tiab] OR cimzia [tiab] OR "golimumab" [Supplementary Concept] OR golimumab [tiab] OR simponi [tiab] → 45 results
- "Abatacept"[Mesh] OR abatacept [tiab] OR belatacept [tiab] OR orencia [tiab] OR CTLA-4-Ig [tiab] OR CTLA4-Ig [tiab] OR CTLA4 Ig [tiab] OR CTLA-4 Ig [tiab] OR nulojix [tiab] OR CTLA4-Fc [tiab] OR CTLA-4-Fc [tiab] OR CTLA4 Fc [tiab] OR CTLA-4 Fc [tiab] → 2 results
- "eculizumab" [Supplementary Concept] OR eculizumab [tiab] OR alexion [tiab] OR soliris [tiab] → 2 results
- "Antibodies, Monoclonal"[Mesh] OR monoclonal antibod* [tiab]) AND (treat* [tiab] OR cure* [tiab] → 161 results
